# Supplementary material for: Diagnosis, Treatment, and Management of Otitis Media with Artificial Intelligence
Source: Diagnostics (Basel). 2023 Jul 7;13(13):2309. doi: 10.3390/diagnostics13132309 (PMC10341128; doi:10.3390/diagnostics13132309)
Supplement: Supplementary file 1 [file diagnostics-13-02309-s001.zip › diagnostics-2403842-supplementary.pdf]

Supplementary

Table S1: ML-related articles for the diagnosis of OM with otoscopy

Year	Author	Classes	Images	Model	Accuracy	Sensitivity	Specificity	Dice
2022	Sandström.J[Error!]	3	273	GoogleNet	95%	93%	100%	—
2022	Chen YC[Error!]	10	2820	InceptionV3	98%	97.90%	99.80%	—
2022	Mao C[Error! Reference]	4	2657	EfficientNet	92.42%	—	—	—
2022	Crowson MG[Error!]	3	661	ResNet, DenseNet	95.50%	—	—	—
2022	Moham KK[Error!]	4	880	CNN, LSTM	100%	100%	100%	—
2022	Choi Y[Error! Reference]	3	6630	EfficientNet	95.32%	95.38%	94.65%	—
2022	Habib AR[Error!]	5	6527	ResNet	74.50%	—	—	—
2022	Zeng J[Error! Reference]	2	2790	ResNet, UNet	81%	—	—	—
2022	Byun H[Error!]	2	1130	CNN	94.10%	91.60%	96.00%	—
2022	Viscaino M[Error!]	4	22000	VGG16	92%	85%	95%	—
2021	Alhud A[Error!]	4	857	CNN	98.26%	97.68%	99.30%	—
2021	Cai Y[Error! Reference]	4	7146	CNN	93.37%	—	—	—
2021	Byun H[Error!]	4	2372	ResNet	97.18%	—	—	—
2021	Crowson MG[Error!]	2	338	ResNet	83.80%	—	—	—
2021	Pham VT[Error!]	4	1012	CNN	95.80%	92.00%	97.60%	0.929
2021	Sundgaard JV[Error!]	3	1336	InceptionV3	86%	—	—	—
2021	Zeng X[Error!]	8	20542	DenseNet	95.59%	—	—	—
2021	Kashani RG[Error!]	2	1179	CNN	90.30%	90.50%	90.10%	—
2021	Tsutsu K[Error!]	5	400	MobileNetV2	77%	88%	84%	—
2021	Basar E[Error!]	2	282	VGG16	88.56%	86.52%	90.25%	—
2021	Cha D[Error! Reference]	6	7500	ResNet	82.03%	—	—	—
2021	Wang W[Error!]	2	100(video)	ResNet	81.70%	83%	80%	—
2021	Binol H[Error!]	4	394(video)	CNN	84.80%	80.20%	94.40%	—
2020	Wu Z[Error! Reference]	3	12305	Xception	97.82%	96.76%	98.37%	—
2020	Khan MA[Error!]	3	2904	DenseNet	94.90%	—	—	—
2020	Cama S[Error!]	3	454	InceptionV2, ResNet	88.10%	—	—	—
2020	Visc MV[Error!]	4	720	SVM	99.03%	98.06%	99.35	—
2020	VT Pham[Error!]	2	1139	CNN	—	—	—	0.895
2020	Habib AR[Error!]	3	233	InceptionV3	76%	—	—	—
2020	Binol H[Error!]	2	73	Random forest	84.60%	—	—	—
2020	Binol H[Error!]	6	136(video)	UNet	—	—	—	0.84
2019	Cha D[Error! Reference]	6	10544	InceptionV3, ResNet	94.20%	93.69%	96.82%	—
2019	Cöme Z[Error!]	4	956	VGG16	99.47%	99.35%	99.77%	—
2019	Livi D[Error! Reference]	14	1366	Google-AutoML-interface	88.70%	86.10%	—	—
2019	Lee JY[Error! Reference]	2	3156	CNN	91.00%	90.50%	92.90%	—
2019	Seok J[Error! Reference]	2	920	Mask R-CNN	91.35%	81.36%	96.03%	—

2018	Tran TT[Error!]	3	1230	CNN	91.41%	89.48%	93.33%	—
2018	Mybu HC[Error!]	5	389	CNN	86.84%	86.80%	96.40%	—
2018	Sena C[Error! Reference]	14	247	CNN	84.90%	—	—	—
2018	Kashani MS[Error!]	2	108	InceptionV3	82.20%	—	—	—
2016	Mybu HC[Error!]	3	562	Decision Tree	80.61%	80.60%	94.40%	—
2015	Chue KS[Error!]	2	1195	SVM	88.50%	89.63%	86.90%	—
2014	CK Shie[Error!]	2	865	Adaboost	88.06%	—	—	—

Table S2: ML-related articles for the diagnosis of OM based on CT, X-ray, OCT and tympanogram

Type	Year	Author	Classes	Images	Model	Accuracy	Sensitivity	Specificity	AUC
CT	2022	Wang ZC[Error! Reference source]	4	973	CNN	90.10%	—	—	0.96
CT	2022	Eroğlu O[Error! Reference source]	4	3093	CNN	95.40%	95.13%	97.92%	—
CT	2022	Takah M[Error! Reference source]	2	4950	MobileNet-V2	81.14%	84.95%	77.33%	—
CT	2022	Duan B[Error! Reference source not]	4	1872	GoogleNet	99%	—	—	—
CT	2020	Wang YM[Error! Reference]	4	1147	Inception-V3	—	83.30%	91.40%	0.92
X-ray	2020	Lee KJ[Error! Reference source not]	2	9988	CNN	90.10%	96.4%	74.5%	0.968
OCT	2022	Monroy GL[Error! Reference]	4	175	Random forest	95.41%	—	—	—
OCT	2019	Monroy GL[Error! Reference]	3	58	Random forest	91.10%	—	—	—
Tympanometry	2022	Sundgaard JV[Error! Reference]	3	1014	CNN	92.60%	92.20%	92.90%	0.97
Tympanometry	2021	Mercha GR[Error! Reference]	4	63	multivariate logistic	94.84%	95.07%	94.64%	0.99
Tympanometry	2021	E. M. Grais[Error! Reference]	2	672	CNN	82%	—	—	0.79
Tympanometry	2020	H. Binol[Error! Reference source]	2	73	Random forest	84.90%	—	—	—

Table S3: Articles for diagnosis of OM using NLP

Year	Author	Classes	Images	Model	Accuracy	Sensitivity	Specificity	F1-score
2021	Binol H[Error! Reference source not]	4	173	NCA	—	87.6	94.4	90.20%
2020	Joshua C[Error! Reference source]	2	2724	NLP	—	83.40%	95.40%	—
2013	Kuruvilla A[Error! Reference]	4	783 patients	Random forest	89.90%	—	—	—
2012	Kuruvilla A[Error! Reference]	4	826	Classifier	84%	—	—	—

Table S4: Articles related to the application of ML in OM surgery

Type	Year	Author	Classes	Images	Model	Accuracy	Sensitivity	Specificity	AUC
Tube	2020	Camalan S[Error! Reference source]	3	454	InceptionV2	88.10%	—	—	0.96
Tube	2019	Devon Livingstone[Error!]	3	734	CNN	84.40%	—	—	—
Tube	2015	Xin Wang[Error! Reference source not]	2	215	SVM	90%	85%	92%	—
Cholesteatoma	2021	Miwa T[Error! Reference source not]	3	312	SSD	—	42.30%	87.50%	—

Table S5: Articles related to the application of ML in the registration of OM surgery.

Type	Year	Author	Classes	Patients	Model	mIOU	mDice
Registration	2022	Ding AS[Error! Reference source not found.]	16	16	ANTs	—	0.56
Registration	2022	Dong B[Error! Reference source not found.]	1	17	FNSegNet	0.764	0.858

Registration	2021	Neves CA[Error! Reference source not found.]	4	150（images）	AH-Net	—	0.82
Registration	2021	Nikan S[Error! Reference source not found.]	8	39	PWD	—	0.86
Registration	2020	Lv Y[Error! Reference source not found.]	3	30	W-Net	—	0.797
Registration	2020	Jeeva S[Error! Reference source not found.]	4	50	Mask R-CNN	0.79	0.96
Registration	2019	Fauser J[Error! Reference source not found.]	9	24	ASMUnet	—	0.663
Registration	2019	Gare BM[Error! Reference source not found.]	1	28	Atlas registration	—	0.76
Registration	2018	Powell KA[Error! Reference source not found.]	6	26	Atlas registration	—	0.7785
Registration	2017	Lu P[Error! Reference source not found.]	1	15	Random forest	—	0.818
Registration	2014	Becker M[Error! Reference source not found.]	8	42	PASM	—	0.802
Registration	2011	Noble JH[Error! Reference source not found.]	7	6	ASM	—	0.745

Table S6: Articles related to the application of ML in the health management of OM [1–98].

Type	Year	Author	Classes	Patients	Model	Accuracy	Sensitivity	Specificity
NLP	2022	Koyam H[Error! Reference source not]	2	105	RF, SVM, kNN	81.50%	—	—
NLP	2017	Dowell A[Error! Reference source not]	6	77582	Software Inference	The Curve of OM morbidity spectrum		
NLP	2012	Szaleniec J[Error! Reference source not]	2	150	NN	84%	—	—
CV	2020	Camalan S[Error! Reference source not]	3	454	InceptionV2	88.10%	—	—
CV	2019	Devon Livingstone[Error!]	3	734	CNN	84.40%	—	—
CV	2015	Xin Wang[Error! Reference source not]	2	215	SVM	90%	85%	92%
